# Observing classical nucleation theory at work by monitoring phase transitions with molecular precision

**DOI:** 10.1038/ncomms6598

**Published:** 2014-12-03

**Authors:** Mike Sleutel, Jim Lutsko, Alexander E.S. Van Driessche, Miguel A. Durán-Olivencia, Dominique Maes

**Affiliations:** 1 Structural Biology Brussels, Vrije Universiteit Brussel, Pleinlaan 2, 1050 Elsene, Belgium; 2 Center for Nonlinear Phenomena and Complex Systems, Université Libre de Bruxelles, Blvd. du Triomphe, 1050 Brussels, Belgium; 3 Instituto Andaluz de Ciencias de la Tierra, CSIC-UGR, Avenida de las Palmeras, 18100 Granada, Spain

## Abstract

It is widely accepted that many phase transitions do not follow nucleation pathways as envisaged by the classical nucleation theory. Many substances can traverse intermediate states before arriving at the stable phase. The apparent ubiquity of multi-step nucleation has made the inverse question relevant: does multistep nucleation always dominate single-step pathways? Here we provide an explicit example of the classical nucleation mechanism for a system known to exhibit the characteristics of multi-step nucleation. Molecular resolution atomic force microscopy imaging of the two-dimensional nucleation of the protein glucose isomerase demonstrates that the interior of subcritical clusters is in the same state as the crystalline bulk phase. Our data show that despite having all the characteristics typically associated with rich phase behaviour, glucose isomerase 2D crystals are formed classically. These observations illustrate the resurfacing importance of the classical nucleation theory by re-validating some of the key assumptions that have been recently questioned.

## Abstract (web summary)

Many nanoscale systems can form ordered microphases through non-classical multistep nucleation. Here, the authors report that glucose isomerase, which is known to exhibit the characteristics of multi-step nucleation in 3D, nucleates along the pathway predicted by classical nucleation theory in 2D.

## Graphical abstract

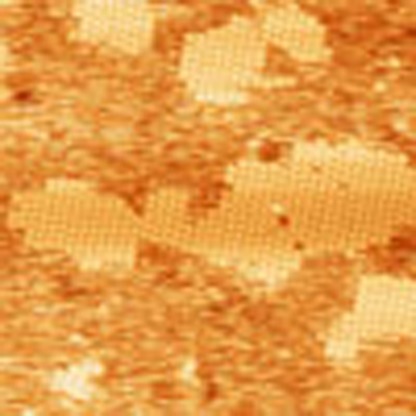

It is perhaps difficult to overstate the importance of nucleation in all branches of natural sciences. A seemingly gratuitous statement, however, the concept of nucleation has been invoked to explain phenomena such as the formation of bacterial appendages, the initiation of neuro-degenerative diseases, earthquakes and more tentatively, the origin of life itself^1^. Indeed, in his classic book on the subject, ‘*Nucleation: Basic Theory with Applications*’^2^, Kashchiev goes beyond the typical examples of (in)organic, protein and colloidal crystallization and widens the discussion with examples as diverse as volcano eruptions, the initiation of divers’ decompression sickness and the formation of black holes to name but a few. In all cases, the importance of nucleation is rooted in the fact that it represents the transition from a stochastic regime dominated by thermal fluctuations to a deterministic regime where growth is thermodynamically driven.

Although the basic theoretical framework known as Classical Nucleation Theory (CNT) was developed more than 50 years ago^2^,^3^,^4^,^5^,^6^,^7^, the field of nucleation is undergoing somewhat of a renaissance as evidenced by numerous recent publications^8^,^9^,^10^,^11^,^12^,^13^,^14^,^15^,^16^,^17^,^18^,^19^,^20^,^21^,^22^, which report on so called non-classical nucleation events that either contest or expand on CNT. These developments have been driven to a large extent by the recent buildout of both experimental and simulation techniques that allow us to observe details of the nucleation process on length and time-scales that were previously inaccessible. Given the wealth of new information, it is not surprising that, despite its well-known simplicity and robustness, numerous short-comings of CNT have now become apparent^23^,^24^,^25^. This has led to renewed interest in the development of theoretical methods that go beyond the simplifying assumptions of CNT such as the use of the capillary approximation to calculate free energies, the assumption of spherical nuclei, the assumption of detailed balance and so on^26^.

Perhaps the most notable outcome of these efforts has been the discovery that the direct, so called single step, nucleation of a new phase is often less favoured than more indirect, multistep processes^9^,^20^,^27^. Indeed, inspired by theoretical and simulation works, experimentalists have searched and found many examples of multistep nucleation (MSN)^8^,^10^,^11^,^13^,^15^,^19^,^21^,^22^ where the system transitions through one or multiple intermediate states, before arriving at the final stable phase. Given the apparent ubiquity of the MSN mechanism as suggested by theory^9^ and numerous experimental examples^8^,^10^,^11^,^13^,^15^,^21^,^22^, the inverse question has become relevant: how common is direct, single-step nucleation of crystalline phases and is there a competition between the two mechanisms in a given system or is each substance predisposed to one and only one nucleation pathway? One of the goals of this contribution is to provide a well-characterized example of crystallization that does nucleate classically in a system known to exhibit the characteristics of the two-step mechanism in other circumstances^28^. Such examples are extremely rare. State-of-the-art techniques utilized for small molecules, such as *in situ* TEM, are well-suited for monitoring the succession of microscopic phases, that is, mapping the nucleation pathways^29^. However, they lack the lateral resolution required to provide a detailed structural characterization of subcritical clusters. As phase identification is based on integrative diffraction techniques, only general statements regarding the nature of the emerging phases can be made (local structural heterogeneity cannot be probed), thereby prohibiting a full dissemination of the actual nucleation mechanism. In this work we employ atomic force microscopy (AFM) to follow the nucleation and growth of 2D protein crystals with molecular precision, allowing us to identify the nucleation pathway and mechanism, simultaneously. For a liquid-to-crystal transition, as is the case here, non-classical nucleation is considered to be those events where local increase in density and crystallinity do not occur simultaneously, but rather sequentially. For the specific system we scrutinized, glucose isomerase 2D crystallization on mica, and we find no evidence that such MSN scenarios might exist despite actively searching phase space for it.

## Results

### Gucose isomerase forms 2D crystals on mica

**Figure 1:**
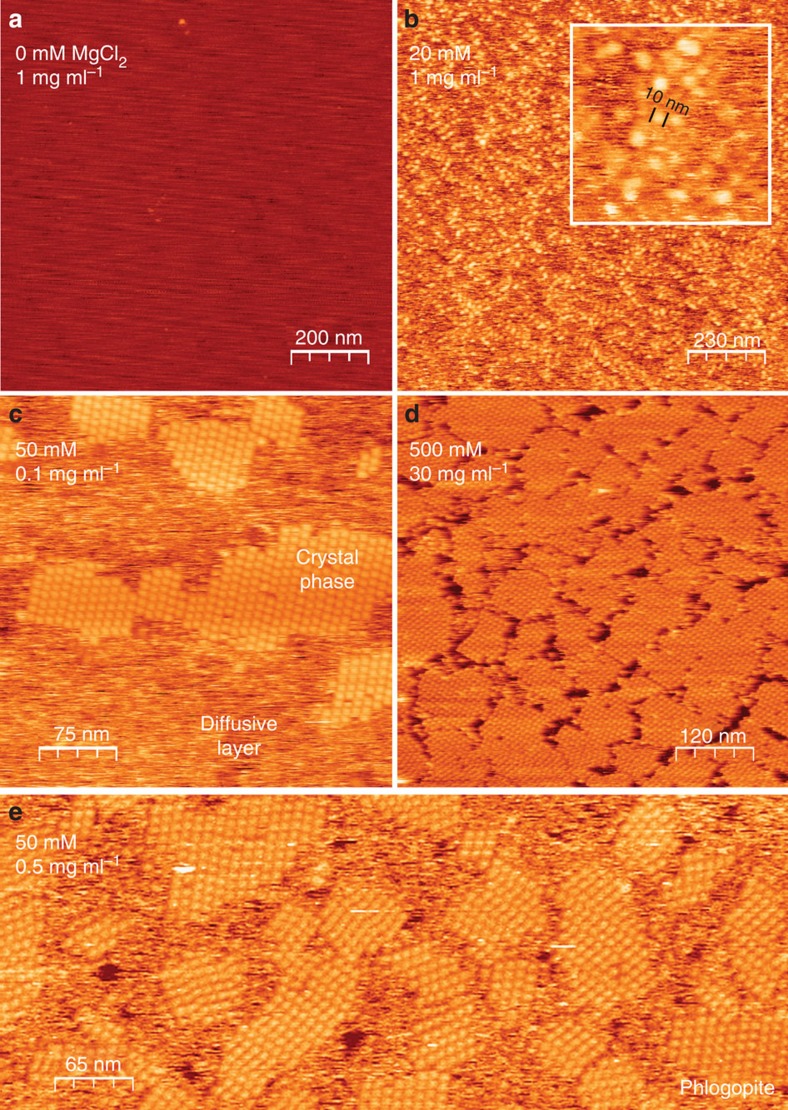
Glucose isomerase 2D crystals on muscovite as a function of [MgCl_2_]. (**a**) 0 mM, no adsorption, (**b**) 20 mM, at random adsorption of glucose isomerase, (**c**) 50 mM, phase separation into a mobile diffusive layer and two-dimensional protein crystals, (**d**) 500 mM even at 30 mg ml^−1^, the crystals do not grow in the vertical direction, (**e**) 2D crystallization using freshly cleaved phlogopite as a substrate.

**Figure 2:**
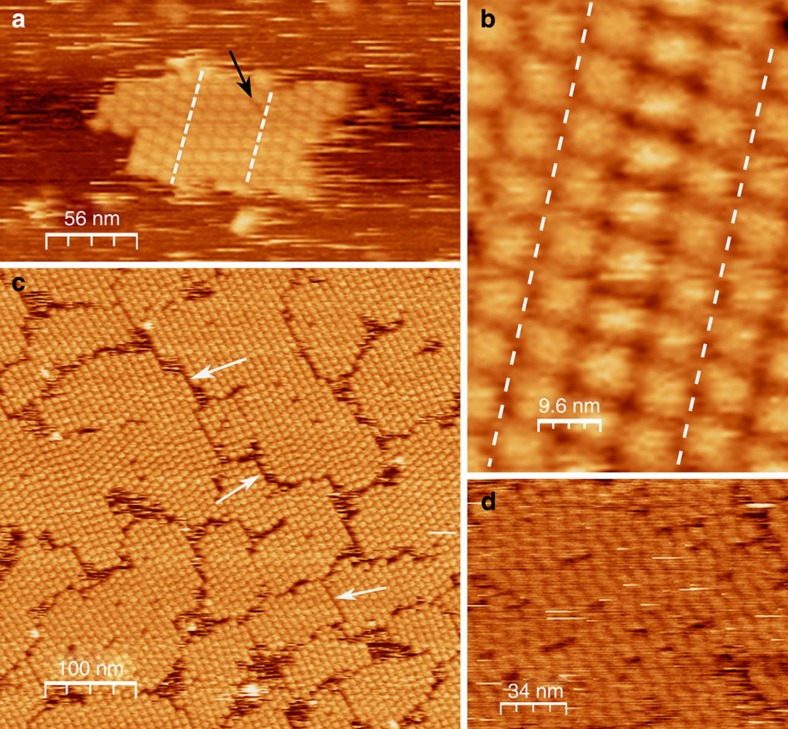
Range of crystallographic defects. (**a**) Twin boundaries within a single cluster (dashed lines) and a vacancy (arrow), (**b**) zoom-in of twin boundaries within a single cluster, (**c**) grain boundaries at the interfaces of independently nucleated clusters (white arrows) and (**d**) plastic crystal phase. The bulk composition was 10 mM Hepes pH 7.0, 50 mM MgCl_2_ (**a**–**c**) and 500 mM MgCl_2_ (**d**) with 0.05 mg ml^−1^ (**a**,**b**) and 0.1 mg ml^−1^ (**c**,**d**) glucose isomerase.

**Figure 3:**
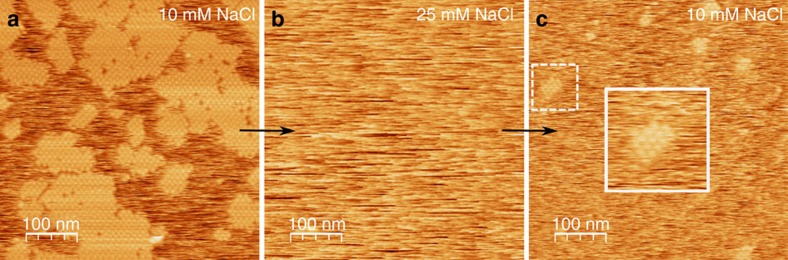
Reversible 2D crystallization as a function of [NaCl]. (**a**) At 10 mM NaCl, 2D crystals are readily formed, which after washing with 25 mM NaCl (**b**) fully redissolve to nucleate anew (**c**) when the [NaCl] is lowered again to 10 mM. Note that the number density of clusters is lower (compared with **a**) due to the shorter delay time between sample injection and the onset of imaging. The bulk composition was 0.05 mg ml^−1^, 10 mM Hepes pH 7.0, 50 mM MgCl_2_ and 10 mM and 25 mM NaCl.

We begin by demonstrating that Hepes buffered glucose isomerase solutions (purified from *Streptomyces rubignosus* and supplied by Macrocrystal Oy—natively present in solution as a dimer of dimers) readily form 2D crystallites when exposed to freshly cleaved muscovite mica (Fig. 1c,d). Crystallographic defects are clearly present: in addition to vacancies, we observe translational lattice disorder resulting in twin boundaries (also see Supplementary Video 1 and Supplementary Fig. 1) within single clusters and grain boundaries at the interfaces between independently nucleated clusters (Fig. 2c). The latter comes from the angular and/or lateral offset between the individual cluster lattices. It is clear that the majority of the 2D crystals are aligned with respect to one another, suggesting that the crystallization is a hetero-epitaxial process. Remarkably, such alignment is completely absent when phlogopite is used as a substrate, a closely related mineral that also belongs to the phyllosilicate group (Fig. 1e). Interestingly, nucleation is only triggered when a threshold concentration of divalent cations is reached in the solution. For instance, for Mg^2+^ we observed at random adsorption for 1 to 20 mM MgCl_2_ (no 2D crystals), whereas 40 up to 500 mM induced rapid nucleation of planar crystalline islands composed of glucose isomerase tetramers (Fig. 1). For higher protein concentrations, a self-assembled monolayer is formed that almost fully covers the mica surface. What drives this adsorption process? At the working pH of 7.0, glucose isomerase has a large net negative charge (estimated to be −17.6*e* using the PropKa 3.1 webserver^30^). Given the negative surface charge of freshly cleaved mica immersed in aqueous solutions^31^, it is reasonable to assume that the divalent cations create electrostatic bridges between the negatively charged protein side chains and the oxygen atoms on the muscovite surface, thus facilitating adsorption. The large number of solvent exposed aspartate and glutamate residues suggests that the molecules will be oriented randomly when adsorbed to the surface (see Supplementary Fig. 2). Both rotational and translational diffusion will be required to align the molecules into the crystalline lattice during nucleation and growth. We make note of two observations that nuance this simplified static picture. First, the propensity of the divalent cations to induce nucleation seems to follow a Hofmeister series dependence^32^. At a fixed salt concentration of 50 mM, we only observed 2D crystals using Ca^2+^ and Mg^2+^, whereas Mn^2+^ and Ni^2+^ did not lead to any discernible protein adsorption on the mica surface (see Supplementary Fig. 3). Zn^2+^ and Cu^2+^ were also tested but no *in situ* AFM observations could be made because these ions trigger rapid protein aggregation within the bulk liquid on mixing with the glucose isomerase stock solution. Typically, such ion-specific effects are framed within the discourse of the chaotropic/kosmotropic divide^33^. If at play here, it implies that the local water structure—or lack thereof— in the vicinity of the protein and/or mica surface is a key factor in the adsorption process. Second, at low salt concentrations (below 40 mM MgCl_2_) individual glucose isomerase tetramers are readily resolved at the mica/liquid interface (Fig. 1b) due to their long residence times. This demonstrates that the protein molecules have a strong vertical interaction with mica. Interestingly, at higher MgCl_2_ concentrations, this vertical interaction is altered and the molecules switch from being stationary to being mobile surface species (Fig. 1c). Effectively, a wetting layer comprising surface diffusing molecules is created once a critical cation concentration is reached and the mobile admolecules become indiscernible with AFM. After an induction time in the range of minutes, crystalline clusters emerge within this mobile layer that either redissolve or laterally expand once they surpass a critical size. The induction time can be increased by lowering the protein bulk concentration and/or the addition of NaCl. For sufficiently high concentrations of NaCl (for example, 25–50 mM), nucleation is halted altogether (Fig. 3b).

We use static light scattering (SLS) to delineate the relative roles of Mg^2+^ and Na^+^ (see Supplementary Fig. 4 and Supplementary Note 1). The colloidal stability of the salt-free solutions is reflected in the large positive second virial coefficient (*A*_*2*_=2.4 × 10^−7^ mol dm^3^ g^−2^), which is the result of long range electrostatic repulsion between the molecules induced by their net negative charge^28^. Shielding of the surface charges by the addition of 50 mM MgCl_2_ significantly lowers *A*_*2*_ to 4.2±0.4 × 10^−8^ mol dm^3^ g^−2^, which is in correspondence with the emergence of the crystalline phase. Supplementing the 50 mM MgCl_2_ solution with 25 mM NaCl -which is sufficient to inhibit nucleation- has no measurable impact on *A*_*2*_ (4.3±0.3 × 10^−8^ mol dm^3^ g^−2^). The lack of change in *A*_2_ is not surprising given that the ionic strength of the solution increases only slightly (from 0.15 to 0.175 M corresponding to a Debye-Hückel screening length of 0.78 and 0.73 nm, respectively). *In situ* observation reveals that the wetting layer of diffusing glucose isomerase molecules is unperturbed by the presence of NaCl. We therefore conclude that (i) the repulsive term in the pairwise interaction potential dominates the second virial coefficient, (ii) NaCl mainly affects the horizontal protein–protein bonding within the clusters, most likely by disrupting the ionic bridges (see below) through either charge neutralization or inducing repulsive hydration forces^34^. We explore the role of electrostatics further by monitoring the crystallization behaviour as a function of pH (see Supplementary Fig. 5 and Supplementary Note 2). We find a clear upper limit for crystallization: at pH 8.0, no crystalline clusters are detected but the diffusive layer remains present on the mica surface. We postulate that for these charge states (−20.14*e* predicted by propka server) the repulsive component of the interaction potential becomes dominant and the averaged protein–protein interaction becomes too repulsive.

### Sub-, near- and super critical clusters are crystalline

**Figure 4:**
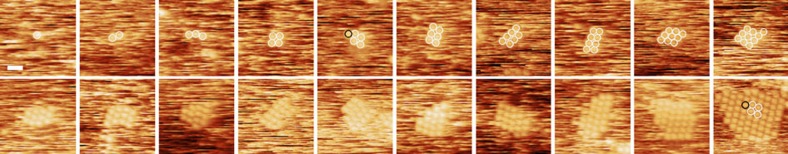
Snapshots of individual clusters ranging in size from 1 to >50 molecules. Remarkably, even the smallest clusters exhibit a highly ordered state commensurable with the lattice of larger sized clusters. However, the local symmetry is not perfect. Note for example the monomer on the left side of the pentameric cluster (black circle): it is out of registry with the lattice defined by the four other molecules (white circles). This displacement is not at random as it coincides with the lattice displacement corresponding to the twin boundaries (to illustrate this, the pattern is overlaid onto the largest sized cluster in the lower right panel). From Fig. 5, we estimate the critical size to be ~20 molecules. Circles in the panels of the upper row (1≤*N*≤10) represent tentative molecular assignments and only serve as a guide for the eye (consult Supplementary Fig. 7 to see more snapshots). Scale bar, 20 nm.

**Figure 5:**
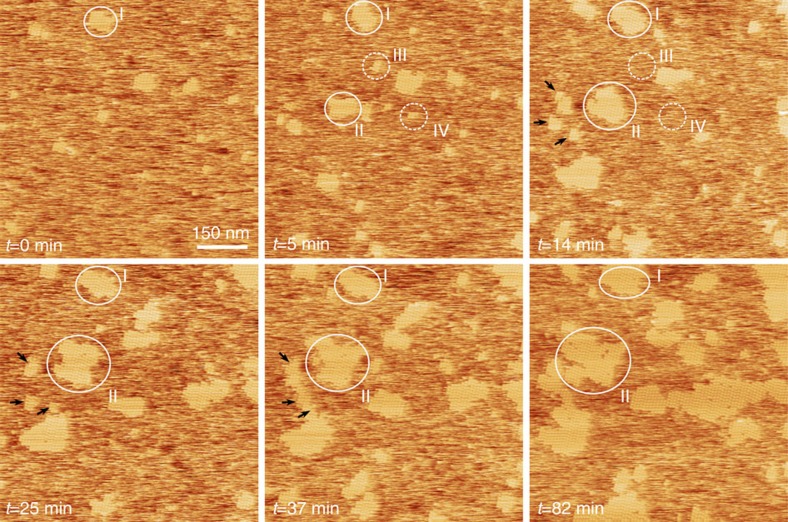
Real-time imaging of the nucleation and growth of 2D glucose isomerase crystals with molecular resolution. The mica surface was initially exposed to a high [NaCl] solution (condition of middle panel of Fig. 3) to wet the surface without inducing nucleation. The AFM liquid cell was subsequently flushed with 0.01 mg ml^−1^, 10 mM Hepes pH 7.0, 10 mM NaCl and 50 mM MgCl_2_ to trigger crystal formation (*t*=0, scan direction for all images upwards). Crystalline clusters rapidly emerge with sizes ranging from 4 to ~40 molecules. Cross-correlating successive images reveal that smaller clusters (III and IV) have a predisposition to shrink or dissolve completely, whereas larger clusters (I and II) tend to amass monomers, notwithstanding temporal fluctuations. This is in accordance with one of the core concepts of classical nucleation, that is, the existence of a critical size that subdivides clusters in groups of either sub- or supercritical. More importantly, it suggests the presence of a local maximum in the cluster size dependence (the relevant order parameter) of the free energy. The presence of such an activation barrier demonstrates that the formation of the 2D crystalline phase occurs through nucleation and not by means of spinodal decomposition. Shortcuts across the nucleation barrier do occur by means of coalescence of independently formed subcritical clusters (black arrows), a pathway outside the scope of CNT.

In the following section, we focus solely on conditions with low protein (<0.1 mg ml^−1^) and/or low NaCl concentrations (1–15 mM). By slowing down the kinetics of the system in this manner, we obtain an experimental window onto the processes that precede and give rise to the nucleation event. Using this approach, we can follow the development of subcritical clusters with molecular precision—a characteristic shared by a few other systems (atoms^35^, colloids^22^,^36^ and proteins^37^,^38^). We find no evidence for the presence of amorphous clusters on the mica–liquid interface, that is, a local density increase is always linked to the alignment of the constituting molecules into a crystallographic arrangement (Fig. 4). This observation holds even for clusters that nucleate and redissolve, which we consider to be sub-critical. This is demonstrated in Fig. 5 that is based on a subset of frames from Supplementary Video 2 in which we follow the system that starts out as randomly diffusing molecules that cluster, cross the critical size and grow as 2D crystals to near surface coverage. Crystalline clusters rapidly emerge with sizes ranging from 4 to ~40 molecules. Cross-correlating successive images reveal that smaller clusters have a predisposition to shrink or dissolve completely, whereas larger clusters, notwithstanding temporal fluctuations, tend to amass monomers indefinitely. Note that we rule out the possibility of simple cluster surface diffusion into and outside the field of view by slow-scan axis disabled imaging on an isolated cluster (see Supplementary Fig. 6). These observations are in accordance with one of the core concepts of classical nucleation theory, that is, the existence of a critical size that subdivides clusters in groups of being either sub- or supercritical. More importantly, it suggests that there is a local maximum in the cluster size dependence of the free energy in this system. The presence of such an activation barrier demonstrates that the formation of the 2D crystalline phase occurs through nucleation and not by means of spinodal decomposition. By measuring the maximum size of clusters, which still tend to dissolve, we estimate the critical size to be ~20 molecules.

The molecular arrangement is clearly scale-invariant, that is, glucose isomerase adopts the same organization in subcritical clusters as in supercritical clusters (Fig. 4b). The distinct absence of a disordered precursor phase preceding the formation of the crystalline monolayer (which is the stable end-state) demonstrates that the system follows a classical one-step nucleation pathway, that is, density and crystallinity increase concertedly. The clear lack of any oligomeric species within the bulk liquid shows that these clusters are energetically stabilized by the cooperative action of both the vertical (mica-protein) and the in-plane (protein-protein) interactions (see Supplementary Fig. 4 and Supplementary Note 1). Formation of crystalline symmetry as a requisite for densification can be rationalized by evaluating the mode of interaction within these clusters. The experimentally obtained lattice parameters of the 2D crystals (determined from images obtained without the use of an O-ring, *u*=8.3 nm, *v*=8.6 nm, *θ*=66°) are in excellent agreement with those derived from X-ray diffraction data on orthorombic 3D crystals (*u*=8.574 nm, *v*=8.574 nm, *θ*=66.32° for the (011) face of the I222 space group). Within this crystallographic arrangement, each monomer interfaces with one nearest neighbour only, repeated four times because of the tetrameric nature of the growth units, through the formation of a salt and hydrogen bridge between Asp80-Arg331 and Arg76-Glu328 (ref. 39). The range of this attractive interaction is in the sub-nanometer range (<0.4 nm) more than one order of magnitude smaller than the hydrodynamic radius of the particles (4.5 nm). From colloid theory, it is well established that systems with a small ratio of range of attraction to particle size (<0.25 (refs 40, 41)) do not exhibit stable dense liquid phases, which would be the 3D analogue of adsorbed amorphous clusters in our 2D case. Hence, for this system, dense liquid phases are expected to be metastable with respect to the crystalline phase. If multi-step nucleation were to occur in this system, the intermediate phases are therefore bound to be metastable. However, their distinct absence in any of our observations rules out this most obvious mode of MSN where the system transitions through distinctly different consecutive phases (be they stable or metastable), a process most closely resembling the Ostwald rule of stages and in complete compliance with classical predictions. This leaves open one final other possibility, which has been dubbed transient two-step nucleation. In transient nucleation, as observed in colloid freezing^42^ and described in theory^43^, liquid-like subcritical clusters are formed first that then gradually solidify into crystalline clusters as the critical size is approached, the important point being that the process is cooperative, characterized by only a single-nucleation event and well outside the scope of CNT. Figures 4 and 5 demonstrate that this is not the case here. Reiterating our previous point, subcritical, critical and supercritical clusters are not disordered and liquid-like but crystalline (see also Supplementary Fig. 7).

## Discussion

Given recent non-classical observations for the 3D case^28^, it is striking to see how well glucose isomerase 2D crystallization fits into the CNT straitjacket. The width of the cluster interface is close to zero, the interior of the cluster is in the bulk state and the cluster dynamics are determined by single molecular attachment and detachment events (see also Supplementary Figs 6 and 8), all of which are well-known CNT restraints. Note that we solely resolve single molecules when they are grouped into a cluster. This clearly shows that only the concerted action of the vertical and horizontal interaction leads to an effective immobilization of the molecules. It is therefore logical (given the short-range interaction) that we observe a defined and not a diffuse cluster interface. Second, the appearance of only one type of dense phase demonstrates that (at least for the conditions tested in this work) molecular arrangements that deviate from a crystalline arrangement are not favoured. This is in agreement with our model of the protein interaction, that is, predominant electrostatic repulsion apart from those orientations commensurable with the lattice leading to attraction. Now, given the characteristics of our system (surface mediated 2D crystallization driven by short range, anisotropic attractive interaction) can one conclude that classical nucleation routes will always be the most efficient crystallization pathway for (hetero)-epitaxial systems? Recent observations on S-layer formation^15^ (2D protein crystals used by pathogenic bacteria as a molecular chain mail for defensive purposes) seems to suggest otherwise. For their system, Chung *et al.*^13,15^ observed a clear delineation between densification and local symmetry breaking, contesting the generality of our observations. There are, however, two important factors that distinguish their system from the more conventional epitaxial case. First, the substrate in their case, a supported lipid bilayer (SLB), is liquid-like (the lateral diffusion coefficient of the lipids in the SLB is 10^−7^–10^−8^ cm^2^ s^−1^)^44^ and, second, the monomers undergo a conformational transformation when relaxing into a crystalline arrangement. Such characteristics are well beyond the scope of CNT and it is therefore not surprising (as also pointed out by the authors) that they lead to non-classical effects.

In a previous work^28^ we have demonstrated that at high concentrations (~100 mg ml^−1^) glucose isomerase can cluster into liquid-like aggregates that are potential precursors of crystalline clusters. Here, due to the wetting effect, nucleation can already be induced at bulk concentrations that are four orders of magnitude lower (~0.01 mg ml^−1^). Although no disordered clusters are present in the bulk for these conditions, one might have expected, given the concentrating effect induced by the external field, 2D amorphous precursors to be present as well. Ultimately, we see no evidence that such a 2D/3D symmetry exists and it seems reasonable to conclude that these disordered dense states will be much higher in energy so that prevalence of one-step nucleation is a logical consequence. Note that the key factors determining the nucleation pathway are the (in)existence of possible intermediate states (primarily a thermodynamic issue^43^), the free-energy barriers separating the various states and the kinematics of thermal fluctuations. Simulation has shown^45^ that even when suitable intermediate states exist, the selection of the pathway depends on a combination of the remaining factors and that there is no simple way to predict which pathway will dominate.

Our AFM data demonstrate a clear alignment of the glucose isomerase clusters with respect to the underlying muscovite lattice. One might speculate that the external field imposed by the substrate in some way penalizes the formation of disordered phases (we rule out possible artefacts by minimizing the applied force with the AFM, Supplementary Fig. 9 and Supplementary Note 3). In other words, is the classical pathway we observe experimentally a result of the templating effect of the substrate? Most works on epitaxial nucleation are primarily concerned with the effect of lattice mismatch and the effect on the critical size and the nucleation rate^46^,^47^. The issue of the templating effect on the nucleation pathway seems not to have been widely investigated until now. However, the absence of any lattice alignment when using phlogopite as a substrate demonstrates epitaxy is not a requirement for nucleation, nor does it greatly influence the nucleation rate (we obtain similar number densities of 2D crystals on both muscovite and phlogopite under otherwise identical conditions, Supplementary Fig. 10). Second, given that the molecules adsorb onto mica in random orientations (the distribution of negative surface side chains does not suggest a guiding mechanism) virtually all relative orientations will be sampled in the diffusive layer, leaving the possibility to form disordered dense states open. From observations in the liquid bulk, however, we know that this type of clustering requires high solute concentrations, concentrations that might simply not be reached in the diffusive layer surrounding the crystalline clusters.

The resurfacing importance of classical nucleation theory is also illustrated in a recent study on heterogeneous nucleation of calcite^48^, a system that, somewhat ironically, has become the archetypal example of multiphase aggregation^10^,^49^. A similar contrasting complexity is found for the protein studied here: we have shown that mesoscopic clusters can play a role in the 3D nucleation of glucose isomerase, suggestive of multi-step nucleation. Our observations on the 2D analogue are therefore particularly revealing: glucose isomerase can, despite having the characteristics typically associated with rich phase behaviour (anistropic interaction^50^, existence of metastable states in the bulk liquid), nucleate classically. These examples emphasize that, although the initial introduction^40^ of the MSN concept has led to great advances in the field, much experimental headway is to be made in understanding what determines the relative efficiencies of the various pathways leading towards the final state. At least already one suggestion has been made to gain control over the pathway selection: Whitelam, on the basis of computer simulation, suggests to tune the relative strengths of the specific and nonspecific interactions^51^, a line of thinking that should certainly be tested experimentally.

## Methods

### Preparation of the protein stock solution

Crystalline suspensions of glucose isomerase purchased from Macrocrystal Oy were dialysed against 10 mM Hepes pH 7.0 and 1 mM MgCl_2_. The dialysis buffer was replaced until the protein solution in the dialysis membrane became clear (full dissolution of the crystals). The protein solution was then concentrated to typically 150–200 mg ml^−1^ using *ε*_280*nm*_=1.042 ml mg^−1^ cm^−1^, filtered using a 0.2-μm cutoff syringe filter and stored at 4 °C.

### Atomic force microscopy

AFM imaging was done in tapping mode using a Nanoscope IIIa multimode AFM (Veeco, Santa Barabara, USA) employing a liquid cell with O-ring to prevent evaporation. Ten-millimetre muscovite discs (Agar Scientific) were glued with two-component epoxy glue onto metal pucks. Before sample loading, the mica was cleaved using sticky tape. Sharpened silicon nitride tips (DNP-S10, Bruker) with a force constant of 0.12 N m^−1^ was used. Flattened images were constructed using WSxM 5.0 (Nanotec^52^) and Gwyddion^53^. To minimize the force applied to the sample while scanning (and counter set point drifts in the system), the set point voltage was continuously adjusted to the lowest level for which tip-sample contact was maintained. Typical drive amplitudes were in the range 100–150 mV. It is important to stress that there is no correlation between the size of the cluster and our ability to resolve the individual molecules. The dominating parameter is the mobility of the molecules, be they isolated or clustered into islands, rather than the size of the cluster.

### Static and dynamic light scattering

SLS data of filtrated Hepes buffered glucose isomerase solutions were collected at 20 °C in 10 mm cylindrical cuvettes at an angle of 90 employing an ALV-CGS-3 static and dynamic light scattering device using a 22 mW He-Ne laser with a wavelength *λ*=632.8 nm. Before sample measurements, detector dark counts were measured and subtracted from the final recorded intensity. The range of concentrations used was 3–10 mg ml^−1^. For SLS, the method outputs the ratio *KC*/*R*_θ_ where *C* is the protein concentration, *R*_θ_=*I*_θ_/*I*_0_ is the Rayleigh ratio of the intensity of the light scattered at angle *I*_θ_ to the incident intensity *I*_0_, *K* is a system constant defined as *K*=(2*πn*_0_/*λ*^2^)^2^(*dn*/*dC*)^2^/*N*_A_, *N*_A_ is the Avogadro number, *n* is the solution refractive index and *n*_0_=1.334 is the refractive index of the solvent at the wavelength of the laser beam. The refractive index increment *dn*/*dC*=0.213 cm^3^ g^−1^ was measured using an Abbe 60-ED refractometer. The ratio *KC*/*R*_θ_ is proportional to the osmotic compressibility


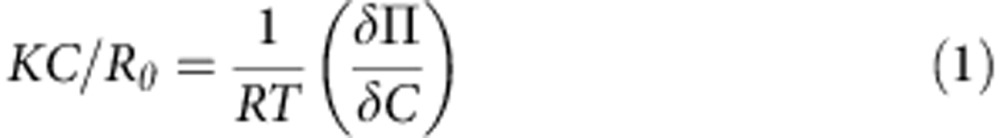


where П is the contribution of the scattering species to the osmotic pressure of the solution, *R* is the universal gas constant and *T* is the absolute temperature. The concentration dependence of the *KC*/*R*_θ_ ratio allows determination of the molecular weight *M*_W_ and the osmotic virial coefficients using ref. 54


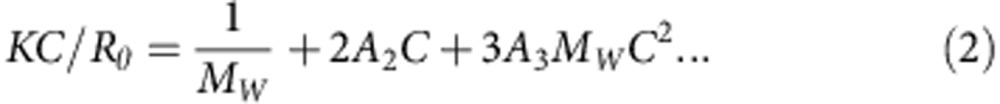


where *A*_2_ and *A*_3_ are the second and third virial coefficients. Given that we operate in the linear regime of the Debye plot, only first order corrections are considered.

Dynamic light scattering was performed contemporaneously with the SLS experiments. Data were collected in a pseudo cross-correlation setup to minimize the contribution of dead time effects and PMT after-pulsing to the recorded signal. The digital correlator outputs the intensity autocorrelation function *g*_2_(*τ*)−1 with *τ* the delay time^55^. This function *g*_2_(*τ*) is connected to the electric field correlation function *g*_1_(*τ*) through the Siegert relation


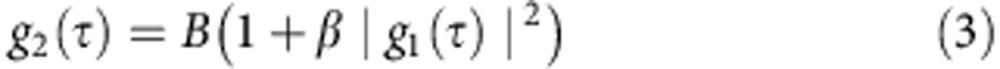


where *B* is the baseline of the correlation function at infinite delay and *β* the function value at zero delay. For a monodisperse solution, *g*_1_(*τ*) is a single exponential decay *g*_1_(*τ*)=exp(−Γ*τ*) with the decay rate Γ=*Dq*^2^ defined by the diffusion coefficient *D* of the particles and the magnitude of the scattering vector 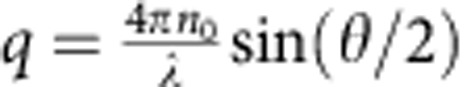 at the scattering angle *θ*.

## Author contributions

The project was conceived by M.S. The atomic force microscopy and the static and dynamic light scattering experiments were performed by M.S. All authors contributed to the data interpretation and the writing of the manuscript.

## Additional information

**How to cite this article:** Sleutel, M. *et al.* Observing classical nucleation theory at work by monitoring phase transitions with molecular precision. *Nat. Commun.* 5:5598 doi: 10.1038/ncomms6598 (2014).

## Supplementary Material

Supplementary Information Supplementary Figures 1-10, Supplementary Notes 1-3 and Supplementary References

Supplementary Movie 1 Time-lapse imaging of a crystalline glucose isomerase self-assembled monolayer reveals the highly dynamic nature of the structure: many molecular detachment and attachment events between successive images reshape the crystalline islands and redefine the grain boundaries. The solution condition is 0.01mg.mL^-1^ glucose isomerase, 10mM Hepes pH 7.0, 50mM MgCl_2_. The delay between two frames is 170s and the observation area is 250x250nm^2^

Supplementary Movie 2 Real-time imaging of the nucleation and growth of 2D glucose isomerase crystals with molecular resolution. During the first 11 frames, the mica was exposed to 0.01mg.mL^-1^, 10mM Hepes pH 7.0 and 50mM MgCl_2_ to pre-wet the surface without inducing nucleation. The AFM liquid cell was subsequently flushed with 0.01mg.mL^-1^, 10mM Hepes pH 7.0, 10mM NaCl and 50mM MgCl_2_ to trigger crystal nucleation (starting from frame 12). Crystalline clusters rapidly emerge with sizes ranging from 4 to ~40 molecules. Cross-correlating successive frames reveals that smaller clusters have a predisposition to shrink or dissolve completely, whereas larger clusters tend to amass monomers notwithstanding temporal fluctuations. This is in accordance with one of the core concepts of classical nucleation, i.e. the existence of a critical size which subdivides clusters in groups of either sub- or supercritical. More importantly, it suggests the presence of a local maximum in the cluster size dependence (the relevant order parameter) of the free energy. The presence of such an activation barrier demonstrates that the formation of the 2D crystalline phase occurs through nucleation and not by means of spinodal decomposition. The scan direction was upwards for all frames, with a delay time of 170s and an scanning area of 750x750nm^2^
