# Supplementary material for: Observing classical nucleation theory at work by monitoring phase transitions with molecular precision
Source: Nat Commun. 2014 Dec 3;5:5598. doi: 10.1038/ncomms6598 (PMC4268696; doi:10.1038/ncomms6598)
Supplement: Supplementary Information — Supplementary Figures 1-10, Supplementary Notes 1-3 and Supplementary References [file ncomms6598-s1.pdf]

# Supplementary Figures

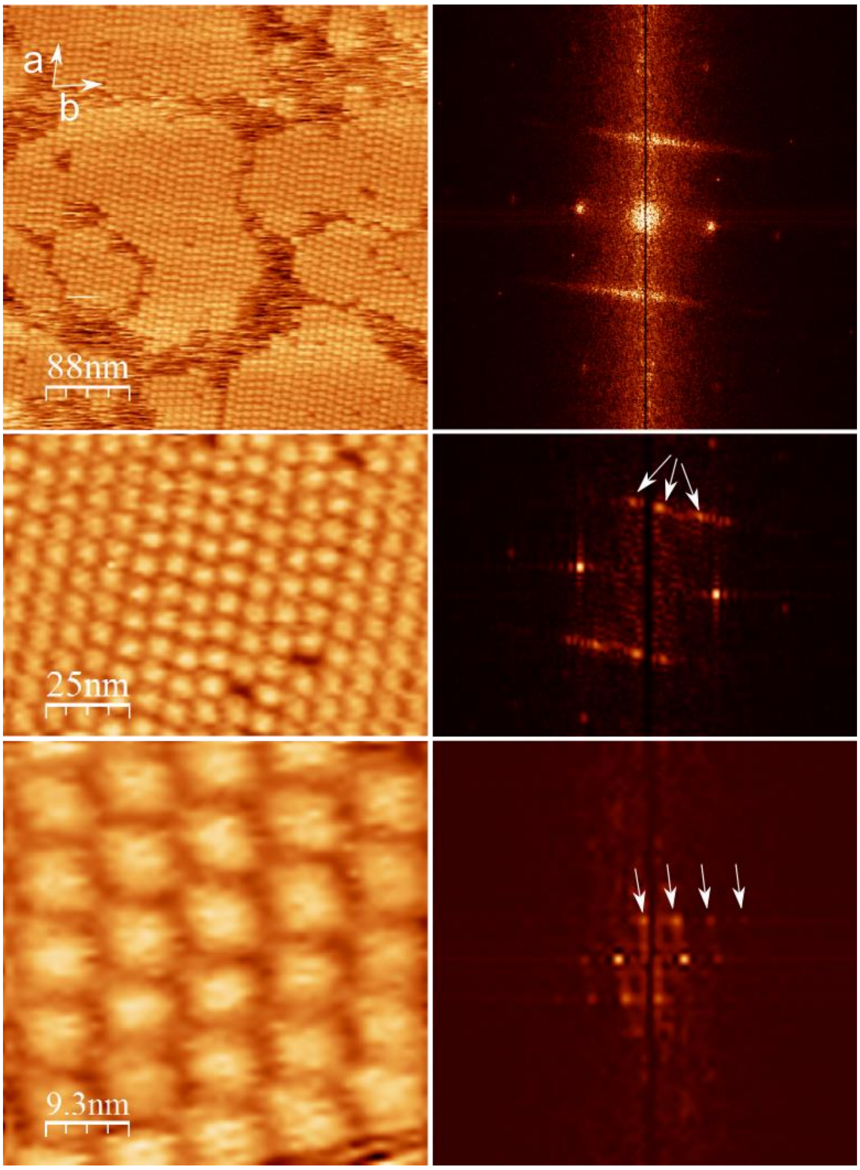


**Supplementary Figure 1: Stacking fault density is direction dependent:** Illustration of the stacking fault multiplicity: lattice disorder is clearly direction specific, gradually zooming into the smallest defect-free domains reveals clear diffraction spots.

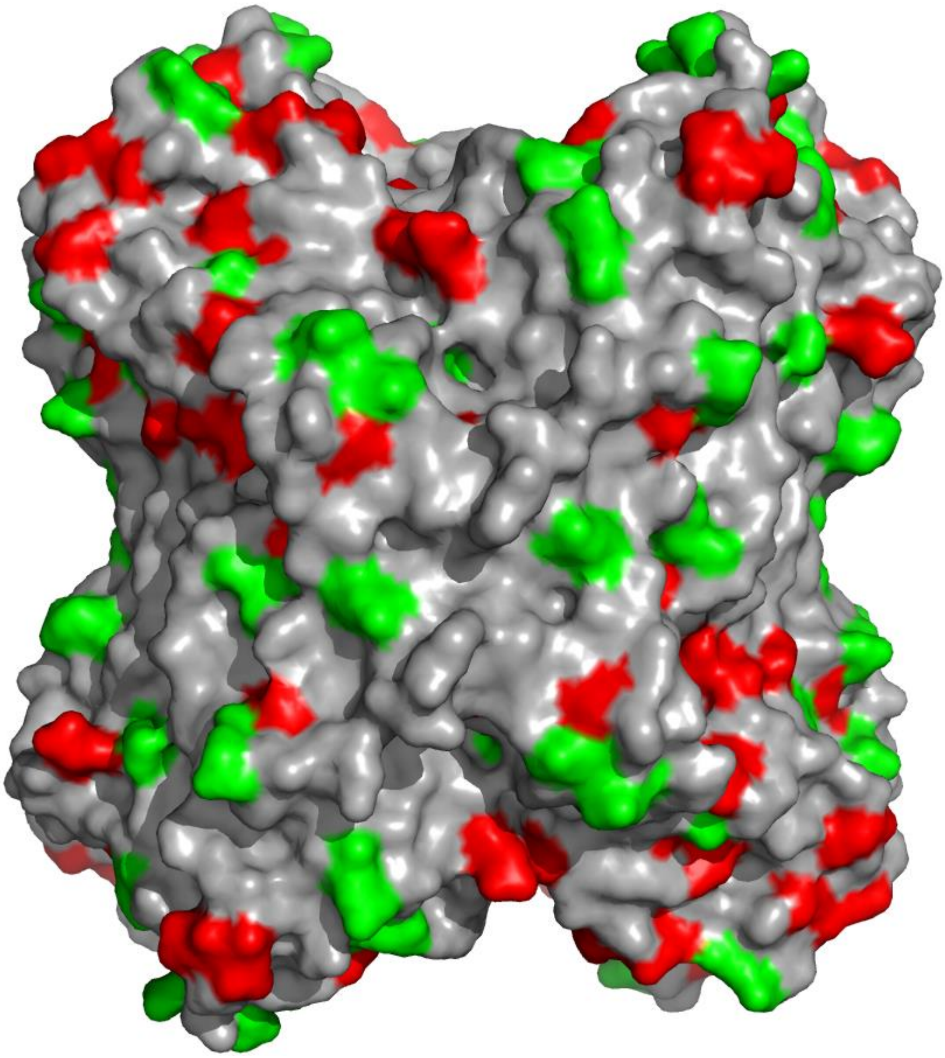

**Supplementary Figure 2: Surface distribution of negatively charged side chains:** Van der Waals surface of a glucose isomerase tetramer: aspartate and glutamate residues are colored red and green, respectively.

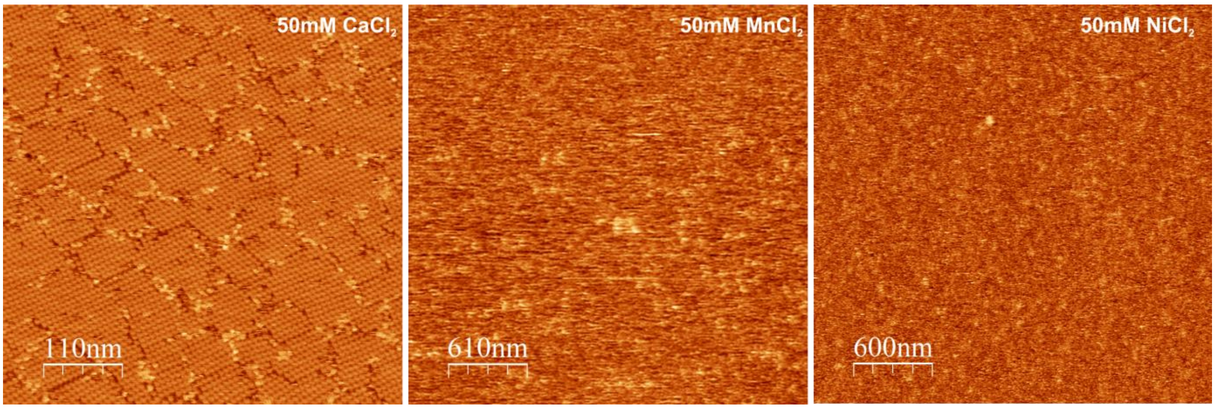


**Supplementary Figure 3**: **Cation-dependence of glucose isomerase 2D crystallization**: AFM height images of the mica-liquid interface for 1mg.mL$^{-1}$ glucose isomerase, 10mM Hepes pH 7.0, 50mM $CaCl_2$ (left panel), $MnCl_2$ (middle panel) and $NiCl_2$ (right panel). Samples containing Ca and Mg readily self-assembled into crystalline monolayers, with protein molecules accumulating at the grain boundaries (left). For Mn and Ni, we did not observe any 2D crystals under otherwise identical experimental conditions (middle and right). We do not rule out that e.g. at higher protein concentration crystallization could still occur, but it does demonstrate that the salting-out propensity of the solution is cation specific.

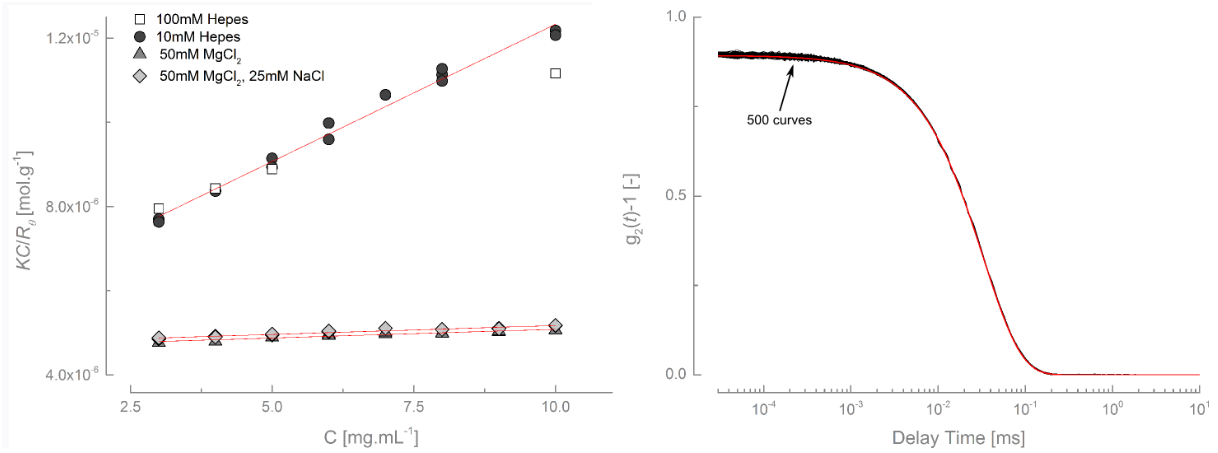


**Supplementary Figure 4**: **Second virial coefficients**: (Left Panel) Debye plots for filtered glucose isomerase solutions with 10mM Hepes pH 7.0 (circles); 10mM Hepes pH 7.0, 50mM $MgCl_2$(triangles); 10mM Hepes pH 7.0, 50mM $MgCl_2$ and 25mM NaCl (diamonds) with linear fits (red lines). The scattering ratios for 100mM Hepes pH 7.0 (squares) taken from Sleutel *et al*[1], are shown as a reference; (Right Panel) Superposition of 500 intensity correlation functions (black lines) of a 0.2 µm filtered 20mg.mL$^{-1}$ glucose isomerase, 10mM Hepes pH 7.0, 200mM $MgCl_2$ solution with the red curve corresponding to a fit using eq.3 in the Methods section of the main article.

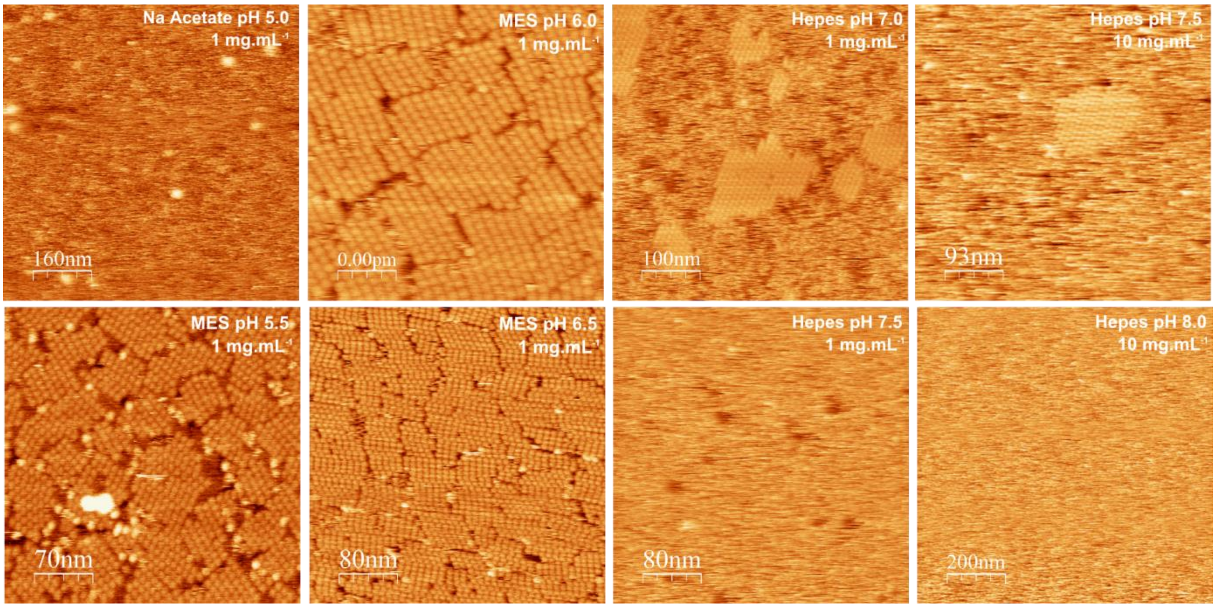


**Supplementary Figure 5: pH-dependence of glucose isomerase 2D crystallization**: AFM height images of the mica-liquid interface for 1 - 10mg.mL$^{-1}$ glucose isomerase, 50mM $MgCl_2$ as a function of pH: 50mM NaAcetate pH 5.0, 50mM 2-(N-morpholino)ethanesulfonic acid (MES) pH 5.5 - 6.5 and 50mM 4-(2-hydroxyethyl)-1-piperazineethanesulfonic acid (HEPES) pH 7.0 - 8.0.

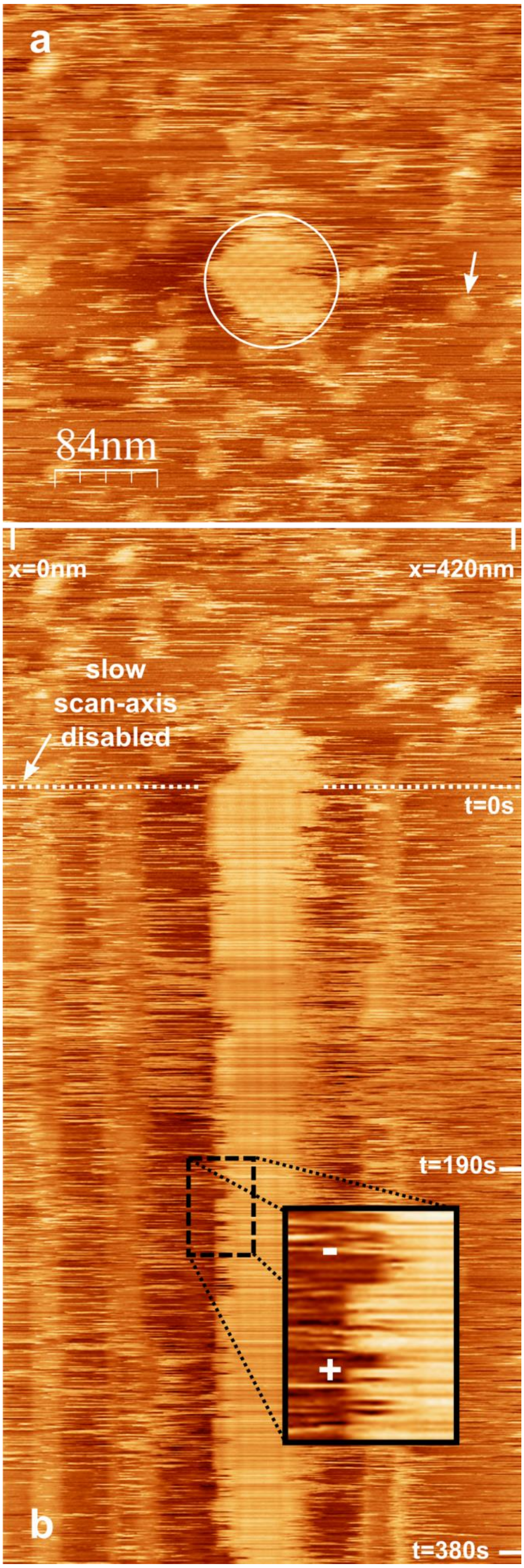


**Supplementary Figure 6: Surface diffusion of 2D clusters is negligible:** (b) Slow scan axis disabled monitoring of a single cluster encircled in (a) (n~50): the temporal evolution of the line-scan centered on the cluster demonstrates that surface diffusion is negligible. Zoom-in of the clusters' step edge (black box) reveals single molecule attachment and detachment events. Note also the presence of smaller clusters without a discernible lattice [arrow in (a)]: their height (3.5-4nm) is not commensurable with the hydrodynamic diameter of a glucose isomerase tetramer (9nm). They must either be a form of Mg-ions (perhaps complexed with Hepes) or unfolded protein. The bulk composition was 0.1mg.mL$^{-1}$, 10mM Hepes pH 7.0, 50mM $MgCl_2$.

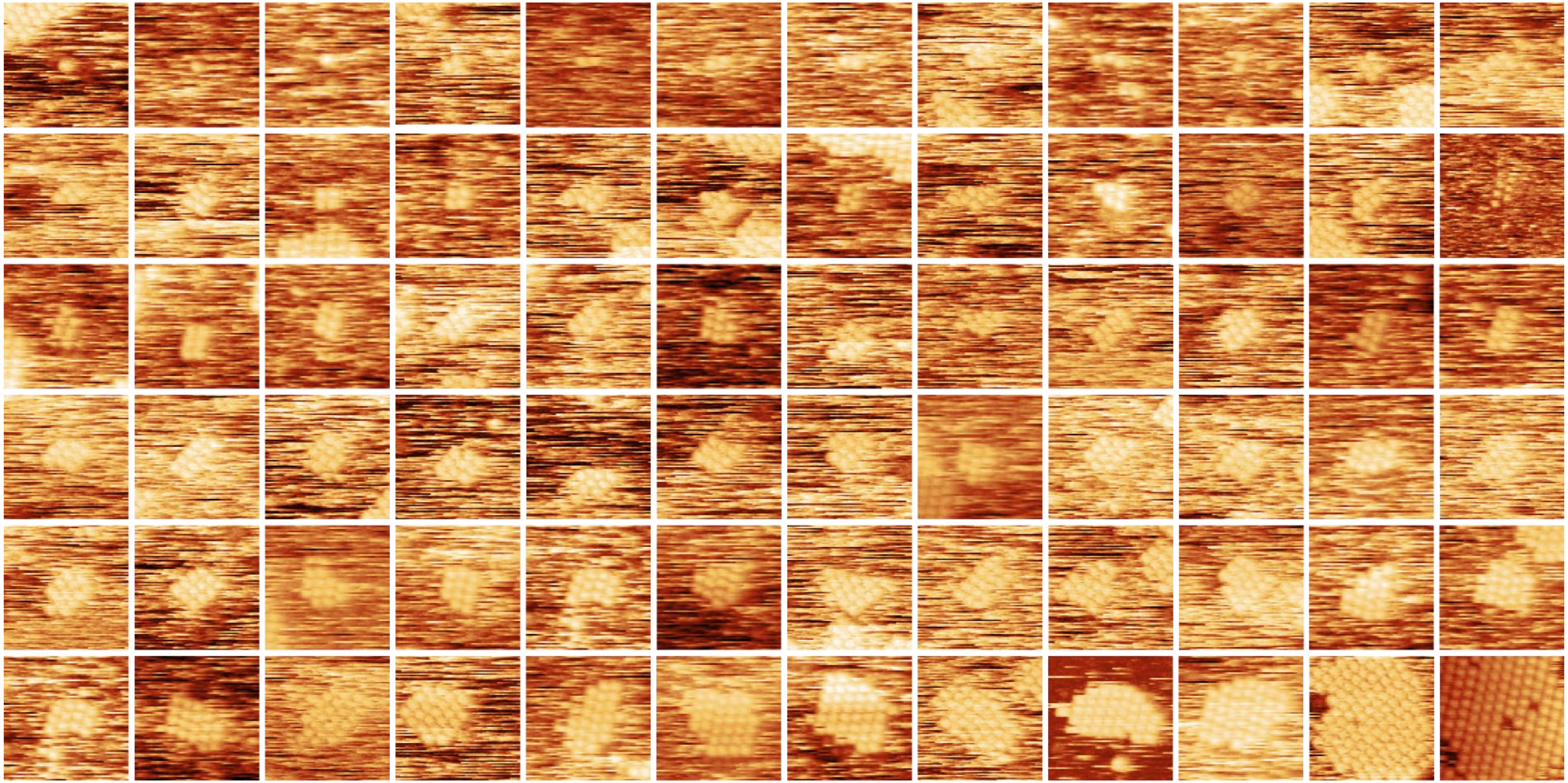

**Supplementary Figure 7**: **Cluster structure as a function of cluster size**: Extended version of Figure 4 in the main text: snapshots of individual clusters ranging in size from 1 to >50 molecules. Note that the orientation of the underlying mica is not constant. Each frame measures 100x100nm$^2$.

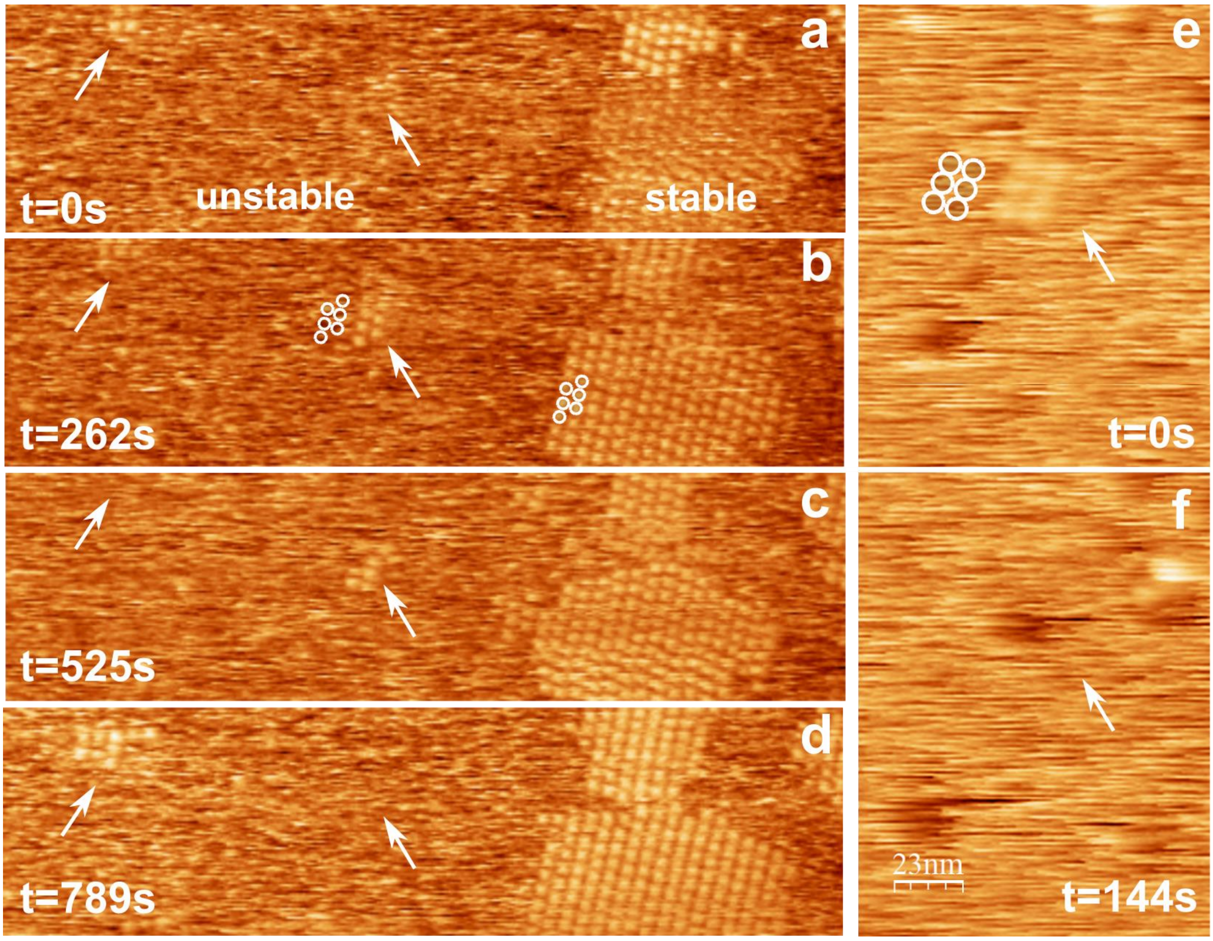


**Supplementary Figure 8: Temporal stability of super- versus subcritical clusters:** (a)-(d) Temporal stability of super- *versus* subcritical clusters: Small clusters (arrows) counting 5 - 8 molecules nucleate seemingly unprompted and quickly redissolve, whereas larger clusters (n>100) are clearly also dynamic but remain stable within the same timeframe. The bulk composition was 0.05mg.mL$^{-1}$, 10mM Hepes pH 7.0, 50mM $MgCl_2$, 0.5% PEG1000; (e)-(f) Another example of a dissolving subcritical cluster with molecules positioned in a clear crystallographic arrangement at 10 mg.mL$^{-1}$, 10mM Hepes pH 7.5, 50mM $MgCl_2$

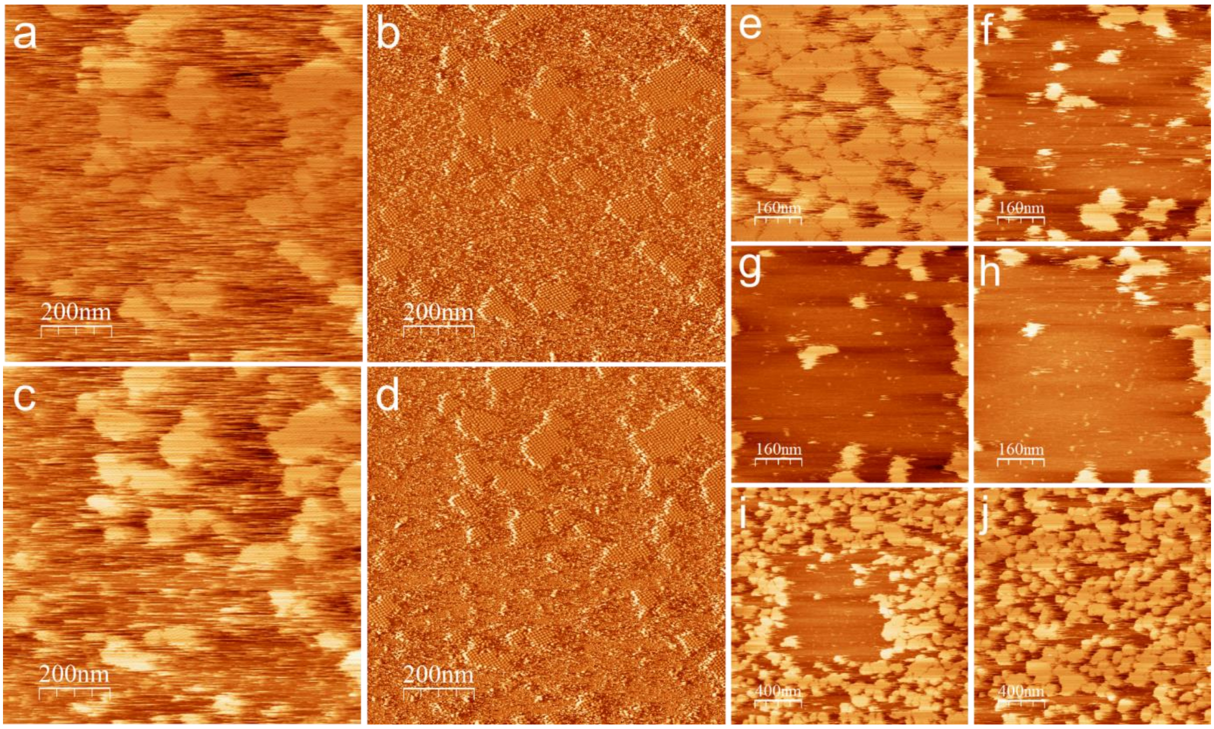


**Supplementary Figure 9: AFM scanning artefact caveats**: AFM scanning artefacts: Two consecutive height (a,c) and deflection (b,d) images using a lower (typical range was 100mV - 150mV) and higher (e.g. 250mV) drive amplitude: the resulting amplitude of the tip oscillation for the higher drive amplitude clearly perturbs the mobile adsorption layer (drive amplitude values are shown as a reference only, they are sample and tip dependent); (e) initial image using a minimal amplitude set point for which tip-sample contact was maintained, (f-h) images recorded after purposefully raising the set point, (i) zoom-out of (e-h) set point reset to its initial value restores the crystalline monolayer (j).

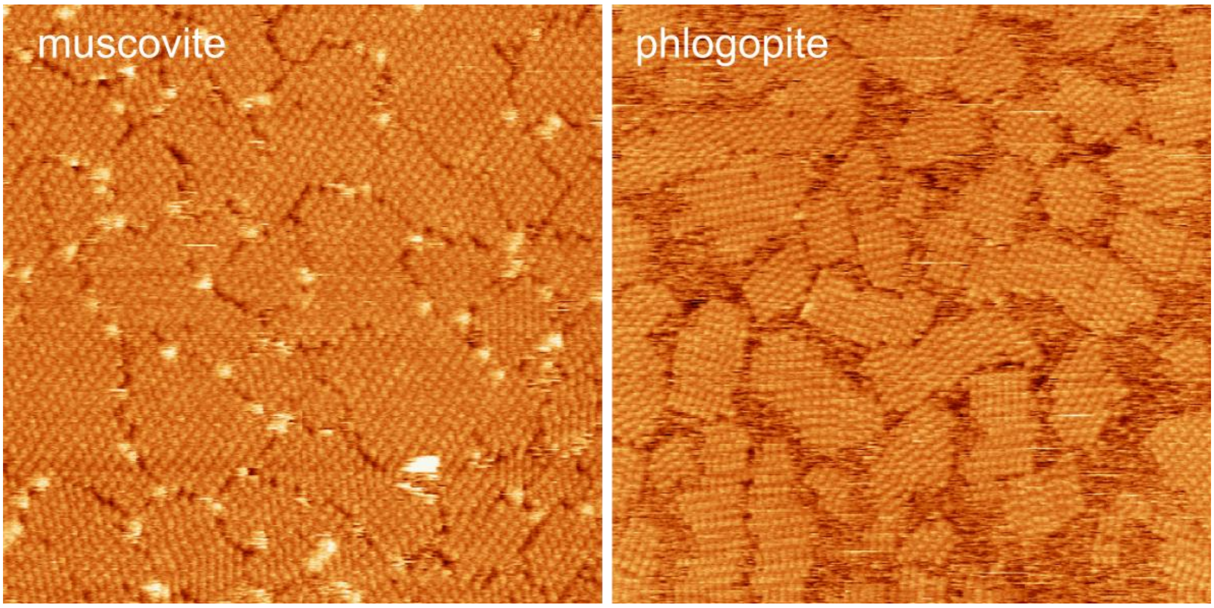


**Supplementary Figure 10: Alignment-free nucleation on phlogopite:** Clear alignment of the crystalline islands when freshly cleaved muscovite is used as a substrate (left), whereas no preferential alignment is observed when using freshly cleaved phlogopite (right). From the comparable grain sizes (in terms of order of magnitude), we infer that the nucleation rate for both systems is similar. The condition used was at 1 mg.mL$^{-1}$, 10mM Hepes pH 7.0, 50mM $MgCl_2$.

# Supplementary Notes

## Supplementary Note 1: Second virial coefficients and monodispersity of the glucose isomerase solution

From a previous study, we know that in 100mM Hepes pH 7.0 solutions, glucose isomerase tetramers strongly repel one another. Not suprisingly, no 2D crystallization is observed for these conditions. However, upon addition of small amounts (<50mM) of divalent cations ($Mg^{2+}$, $Ca^{2+}$) glucose isomerase readily forms 2D crystals. Conversely, supplementing a 50mM $MgCl_2$ buffered solution with 25mM NaCl nucleation is inhibited. Second virial coefficients were determined using SLS for three conditions (Hepes only, supplemented with Mg, supplemented with Mg and Na) to monitor the respective effect on protein-protein interaction. For 10mM Hepes pH 7.0, $A_2$=2.4x10$^{-7}$ mol.dm$^3$.g$^{-2}$; adding 50mM $MgCl_2$ lowers $A_2$ to 4.2x10$^{-7}$ mol.dm$^3$.g$^{-2}$ and supplementing the solution further with 25mM NaCl has virtually no impact on $A_2$, which is 4.3x10$^{-7}$mol.dm$^3$.g$^{-2}$ (Supp.Fig.4 left panel).

One could hypothesize that the 2D glucose isomerase crystals observed on the mica-solution interface are initially formed in the solution and simply sediment onto the substrate. Extended DLS monitoring (500 correlograms collected in a 20h time-frame) reveals that this is not the case. Excellent fits are obtained using a single exponential decay, demonstrating that the filtered glucose isomerase solutions are highly monodisperse (Supp.Fig.4 right panel).

## Supplementatry Note 2: pH-dependence of glucose isomerase 2D crystallization

From experiments described in the main text (see Fig.3 of the main manuscript), we know that monovalent cations (Na) can reduce/inhibit 2D crystallization, which suggests that electrostatics play an important role in the condensation process (as is also evident from the salt-bridges that constitute the lattice contacts of glucose isomerase). Interestingly, addition of Na only perturbs the formation of the crystalline state, the diffusive adsorption layer remains present at concentrations where nucleation is completely inhibited (25mM NaCl). An alternative approach to change the electrostatic component of the protein-protein interaction potential is by means of changing the pH. By increments of 0.5, we increase the pH from 5.0 to 8.0 (see Supp.Fig.5, also note that the lower pH-limit is set by protein stability restraints, below 5.0, glucose isomerase readily denatures). The operational range of Hepes is from 6.8 to 8.2, so other buffering agents (we've opted for NaAcetate and 2-(N-morpholino)ethanesulfonic acid) are required to reach the lower pH values, keeping all other components (and their concentration) constant.

No 2D crystallization is observed at pH 5.0 and 8.0, limiting the crystallization slot to 5.5 - 7.5, although at pH 7.5 we did have to increase the protein concentration from 1 to 10 mg.mL$^{-1}$ to induce nucleation. We remark that the goal of these experiments is to identify the relevant order parameters, not to determine an exhaustive (multi-dimensional) phase diagram. What processes determine the upper and lower pH-limit of the crystallization slot? At higher pH values, the net surface charge of a glucose isomerase tetramer becomes increasingly more negative (the propka webserver predicts a protein charge of -20.14e at pH 8.0). Interestingly,

at this higher pH value, the diffusive layer is still present, suggesting that the vertical protein-mica interaction is still attractive. We postulate that for these charge states, the repulsive component of the interaction potential (due to the like-charge) becomes dominant and the in-plane protein-protein interaction becomes repulsive. The lower limit is perhaps less evident. The predicted pKa values of the charged amino acids (Asp80-Arg331 and Arg76-Glu328) partaking in the lattice contact are 4.16, 12.45, 12.37 and 4.65, respectively, well outside the empirical crystallization slot - disruption of the ionic bonds due to (de)protonation should therefore not be the main inhibitory cause. Alternatively, perhaps the reduced structural stability of the growth species (at this lower pH) is a factor that is limiting the crystallization.

## Supplementatry Note 3: AFM scanning artefact caveats

Tapping mode AFM microscopy relies on measuring the direct (albeit intermittent) interaction between the sample and a spring-mounted tip to construct a digital image of the surface topography. This *in situ* mechanical mode of imaging leads to lateral resolution values for biological specimens that can be two to three orders of magnitude smaller than the optical diffraction limit. But to achieve such a resolution, caution is advised. As is well-known, if the tip-sample force is too high, one can perturb or even damage the sample. Below we demonstrate both possibilities. In Supp.Fig.9a-d, two consecutive images (both the amplitude as well as the deflection image) are shown using a lower (a,b) and a higher (c,d) drive amplitude value. Closer inspection of the deflection images (Supp.Fig.9c,d) reveals that the diffusive adsorption layer is disturbed for the higher drive amplitude case, revealing the mica surface underneath.

Even more extreme is the example shown in Supp.Fig.9e-j. Starting from Supp.Fig.9f, the amplitude set point was purposefully raised leading to rapid destruction of the crystalline islands. The zoom-out in Supp.Fig.9i clearly shows the localized nature of the damage, which is readily corrected by renewed nucleation and growth upon lowering the amplitude set point to its initial value. Performing such a series of scanning tests allows us to establish a safe operational window where we can rule out any disturbing effect of the scanning tip.

# Supplementary Reference

1. Sleutel, M. & Van Driessche, A. E. S. Role of clusters in nonclassical nucleation and growth of protein crystals. Proc. Natl. Acad. Sci. U. S. A. 546–553 (2014).
